# Extending trust to immigrants: Generalized trust, cross-group friendship and anti-immigrant sentiments in 21 European societies

**DOI:** 10.1371/journal.pone.0177369

**Published:** 2017-05-08

**Authors:** Meta van der Linden, Marc Hooghe, Thomas de Vroome, Colette Van Laar

**Affiliations:** 1 Centre for Citizenship and Democracy, University of Leuven, Leuven, Belgium; 2 European Research Centre on Migration and Ethnic Relations, Utrecht University, Utrecht, The Netherlands; 3 Center for Social and Cultural Psychology, University of Leuven, Leuven, Belgium; Western Sydney University, AUSTRALIA

## Abstract

The aim of this study is twofold. First, we expand on the literature by testing whether generalized trust is negatively related to anti-immigrant sentiments in Europe. Second, we examine to what extent the relation between generalized trust and anti-immigrant sentiments is dependent upon cross-group friendships. We apply multilevel linear regression modeling to representative survey data enriched with levels of ethnic diversity covering 21 European countries. Results show that both generalized trust and cross-group friendship are negatively related to anti-immigrant sentiments. However, there is a negligible positive relation between generalized trust and cross-group friendship (*r* = .10), and we can clearly observe that they operate independently from one another. Hence, trusting actors are not more likely to form more cross-group friendships, and cross-group friendship do not lead to the development of more generalized trust. Instead, the findings show that generalized trust leads immigrants too to be included in the radius of trusted others and, as a consequence, the benign effects of generalized trust apply to them as well. We conclude that the strength of generalized trust is a form of generalization, beyond the confines of individual variations in intergroup experiences.

## Introduction

Most studies have consistently noted that the ethnic-cultural majority in Europe is anxious about immigration and its impact on society [1–7]. Constrict theory [8] has hypothesized that exposure to ethnic diversity leads people to withdraw from others and social life to the extent that they trust others less and intergroup relations deteriorate. Contact theory [9–11], however, proposes a more optimistic view by showing that people living in ethnically diverse contexts have more contact with immigrants which increases trust and improves intergroup relations. Although the two theories have different predictions regarding the consequences of ethnic diversity for anti-immigrant sentiments, both perspectives point toward the importance of trust in others in establishing more positive intergroup relations. Particularly generalized trust is considered an essential building block for social cohesion in communities [12–14]. Generalized trust is defined as the basic expectation that most people can be trusted [15]. Generalized trust is an abstract outlook toward people in general, encompassing a wide circle of unfamiliar others (e.g. strangers, fellow citizens, foreigners). As it can be assumed that attitudes toward familiar others will be mainly positive, generalized trust is expected to have a positive impact primarily on attitudes toward those who are unfamiliar and often have different background characteristics than the actor [14]. This has been confirmed by recent studies which have linked generalized trust to increased levels of tolerance [16] and lower levels of religious and racial prejudice [17].

However, the literature remains divided as to how this negative relationship between generalized trust and anti-immigrant sentiments can be explained, or more specifically how dependent this relation is upon intergroup contact. Hence, it is unclear whether generalized trust is an evaluation grounded in concrete experiences or whether generalized trust is a personal predisposition that is innate or developed early in life [15,18]. Empirical research lends some support to the idea that intergroup contact builds trust, which in turn reduces negative intergroup attitudes [19–21]. However, this notion has mostly been supported for other types of trust, such as outgroup (or intergroup) trust [22], but might not be replicated for generalized trust in particular [14], this is the focus point of the current study. Outgroup trust and generalized trust are two very different concepts: Outgroup trust is an attitudinal measure of trustworthiness of a particular outgroup [23,24], while generalized trust is a general disposition to what extent one trusts (unfamiliar) others in general [14,25]. It is therefore unlikely that findings of studies that include outgroup trust simply generalize to hypotheses including generalized trust. The extent to which generalized trust is linked to intergroup contact has important implications for the role of generalized trust in explaining anti-immigrant sentiments. If generalized trust is indeed not experience based [14,25], the robustness of generalized trust to outside influences makes it a crucial factor for peaceful coexistence. Generalized trust would operate as a notion of a benefit of the doubt that should be extended toward all potential interaction partners, whether or not interaction has already taken place [26,27].

In sum, the aim of the current paper is to a) expand existing literature by examining to what extent generalized trust is negatively related to anti-immigrant sentiments, and b) examine whether this relationship is dependent upon positive intergroup contact, i.e. cross-group friendships. Immigration as discussed in this study includes all people who have migrated from their country of birth to their current country of residence. We contribute to the literature on anti-immigrant sentiments in the following ways. First, most studies on the relation between generalized trust and contact did not directly measure contact but included proxy variables such as voluntary engagement or membership [18]. These studies did not find evidence for a relation between contact and generalized trust [28,29]. However, these results remain unpersuasive as voluntary engagement does not necessarily imply intergroup contact. Moreover, studies that have included a direct measure of intergroup contact only focused on contact with familiar others or ingroup members. Glanville, Andersson and Paxton found that people who relatively often engage in various forms of socializing with friends, relatives, and neighbors have higher levels of generalized trust [30]. However, it is unclear whether this finding holds for intergroup contact with people who have different background characteristics such as immigrants. Hence, the current study does include a direct measure of intergroup contact between ethnic-cultural majority members and immigrants. Second, studies on generalized trust and contact that have measured contact only focus on the *frequency* of contact, but do not directly measure the *quality* of intergroup contact [18,20]. This is surprising since especially positive intergroup contact has been hypothesized to aid the development of trust [31]. Therefore, we take into account the role of cross-group friendships for the relation between generalized trust and anti-immigrant sentiments. Cross-group friendships have been proven to be more effective in improving intergroup attitudes than other forms of intergroup contact [32–34]. Hence, cross-group friendships are likely to be a particularly effective form of intergroup contact to possibly facilitate a negative relationship between generalized trust and anti-immigrant sentiments. Third, we use multilevel analysis on representative individual-level survey data (e.g. cross-group friendship, generalized trust, and anti-immigrant sentiments) from the European Social Survey covering 21 countries enriched with a society-level indicator of ethnic diversity (i.e. the percentage of immigrants in a country). With this approach, we answer the call for multilevel approaches to place the study of anti-immigrant sentiments in real-world contexts that include actual daily experience with cultural diversity [35,36].

### The inclusion of immigrants in the circle of trusted others

Generalized trust can be considered “as one of the most important synthetic forces within society” (p. 393–394) [13,37] with a multitude of positive outcomes: “Virtually all research suggests that generalized trust has beneficial effects on individuals, communities, the workplace, institutions and, indeed, nations. Trust makes people healthier, happier, and more hospitable” (p. 462) [38]. The extent to which these benign effects of generalized trust apply to attitudes toward immigrants as well depends on whether immigrants are included in our trusting judgments, which has been debated. In other words, generalized trust must apply to some extent to immigrants for the negative relation between generalized trust and anti-immigrant sentiments to occur. Fukuyama was among the first to distinguish between the level and the radius of generalized trust [39]: How wide a circle do people imagine as “most people” when they answer the standard trust question “Generally speaking, would you say that most people can be trusted or that you need to be very careful when dealing with people?” [40]. In their pioneering work, Delhey and colleagues examine the radius of generalized trust across 51 countries and revealed that in most countries generalized trust both predicts ‘ingroup trust’ (i.e. trust in family, neighbors, and people known personally) as well as ‘outgroup trust’ (i.e. trust in people met for the first time, people from a different religion and nationality). They conclude that “in most places, respondents do imagine a wider circle of people when answering the standard, unspecified question” (p. 800) [40]. A similar study was performed by Van Hoorn [41] who showed that especially individualistic cultures are characterized by a broader radius of trust, which is applicable to the European societies studied in the current research.

Based on these studies of the radius of generalized trust, Reeskens [16] explored the degree to which trusting individuals are actually outward reaching toward marginalized societal groups. Reeskens found a negative relation between generalized trust and social distance with regard to cultural minorities, with social distance examined in a rather broad set of social groups, namely people of a different race, immigrants and foreign workers, Muslims, Jews, and people with large families. Ekici and Yucel [17] also found a positive relationship between generalized trust and a similar measure of social distance, namely willingness to live next to a neighbor from a different race or religion. Even though both studies do not focus specifically on anti-immigrant sentiments, we expect that it is likely that generalized trust includes immigrants as well. We hypothesize a negative relationship between generalized trust and anti-immigrant sentiments in European societies (H1).

### The role of cross-group friendship for the relationship between generalized trust and anti-immigrant sentiments

If there is indeed a negative relationship between generalized trust and anti-immigrant sentiments, how does this relationship come about? For other types of trust, such as outgroup trust, there exists empirical support for the conditional role of intergroup contact for building trust between groups in order to improve relations between groups [19–21]. In other words, intergroup contact is argued to affect people’s willingness to trust members of other groups extending the prejudice-reducing effect of contact [31]. However, for generalized trust, the role of contact is less clear. There is an ongoing debate about whether generalized trust is built from personal experiences, such as intergroup contact, or whether generalized trust is a more fixed predisposition [18,42]. Based on the literature on the origins of trust, we can consider two general perspectives on the role of intergroup contact for the relation between generalized trust and anti-immigrant sentiments, which have been summarized well by Freitag and Traunmüller [15]: The first theoretical perspective considers trust an evaluation of a person’s “social environment and therefore grounded in *concrete experiences of trustworthiness* [emphasis in the original] in social interaction”. The second perspective considers trust “a general propensity either innate or learned early in life and is thus primarily a *personal predisposition* [emphasis in the original]” (p. 787). Concrete experiences of trustworthiness as described in the first theoretical perspective have mostly been found to shape trust in a particular outgroup that does not necessarily extend to outgroups with other demographic characteristics [19–21]. Generally, the second theoretical perspective is considered more relevant for generalized trust, because trust in most people from a variety of different groups—even strangers from outgroups one has never met—cannot completely rest on personal experience. Uslaner argues that “we don’t learn to trust ‘most people’ by evidence. We can’t meet most people. Nor can we generalize from the people we know to ‘most people’”, so generalized trust is not “a judgment that others are trustworthy, but rather that we should treat strangers *as if they were trustworthy* [emphasis in the original]” (p. 7) [14]. Uslaner considers generalized trust as an optimistic worldview that is likely inherited through socialization rather than acquired through an accumulation of experiences: It is “a general outlook on human nature and *mostly* [emphasis in the original] does not depend on personal experiences or upon the assumption that others are trustworthy” (p. 17) [25].

Based on above arguments we expect that cross-group friendships and generalized trust are not related (H2). In addition, we also test for a moderation effect, because, even if cross-group friendship and generalized trust are not correlated, generalized trust could still moderate the association between cross-group friendships and anti-immigrant sentiments. However, again based on the above arguments, we do not expect that the negative relation between generalized trust and anti-immigrant sentiments is dependent upon cross-group friendships (H3).

### The consequences of ethnic diversity for anti-immigrant sentiments

Ethnic diversity sets the stage for the above mentioned hypotheses on the role of generalized trust and cross-group friendships for anti-immigrant sentiments. The consequences of ethnic diversity for social cohesion have been sharply debated. The robustness of generalized trust has been the subject of Putnam’s controversial constrict theory [8], which hypothesizes that “[. . .] people living in ethnically diverse settings appear to ‘hunker down’—that is, to pull in like a turtle” (p. 149). This would in turn deteriorate generalized trust and the quality of native-immigrant relations. A recent meta-analysis scrutinized the results of 90 studies that examined ethnic diversity in different geographical areas on different forms of social cohesion. The result has been ‘a cacophony of empirical findings’ [43]. Whether ethnic diversity is harmful to native-immigrant relations is in part determined by whether or not studies included measures of intergroup contact [22]. Studies that have measured ethnic diversity and ethnic or racial attitudes—but not individual-level intergroup contact—appear to treat context-level diversity as a ‘proxy’ for contact, and, thus, presume that individual-level contact variables are not needed; this subset of papers tends to show more pessimistic consequences of diversity. Studies that have included measures of intergroup contact generally found that intergroup contact cancelled out any direct negative consequences of ethnic diversity on racial attitudes or trust [20,44]. However, these studies focused on other types of trust than generalized trust.

To our knowledge, there is only one study that has included measures of intergroup contact and generalized trust when examining the relation between ethnic diversity and anti-immigrant sentiments [45]. In their multilevel analysis of local communities in Belgium, Hooghe and De Vroome found that citizens living in ethnically diverse communities do not hold more negative attitudes toward immigrants. The current research further adds to this study by a) taking a cross-national approach to examine whether this finding holds for 21 European societies, and b) examining the consequences of ethnic diversity with and without inclusion of cross-group friendship and generalized trust, to show the potential differences in strength regarding its relation with anti-immigrant sentiments. We hypothesize that ethnic diversity does not predict an increase in anti-immigrant sentiments (H4).

## Method

### Data and respondents

The first wave of the European Social Survey (ESS-1) from 2002 was used. The ESS is a cross-national survey with representative samples covering 22 different European countries. We chose the ESS-1, because it measures a broad spectrum of anti-immigrant attitudes and it is one of the few large-scale cross-national surveys that include a measure for intergroup contact. The ESS is carried out with hour-long face-to-face interviews focusing on topics such as immigration, citizenship and socio-political issues. Respondents were selected through strict random probability sampling and the survey was translated to fit each country’s native language through rigorous translation protocols [46]. The following 22 countries participated in the ESS-1: Austria, Belgium, the Czech Republic, Denmark, Finland, France, Germany, Greece, Hungary, Ireland, Israel, Italy, Luxembourg, the Netherlands, Norway, Poland, Portugal, Slovenia, Spain, Sweden, Switzerland, and the United Kingdom. In total, 42,359 respondents participated. Israel was excluded from the analysis due to the unique nature of the immigration context in that country. Moreover, because we were primarily interested in the host population’s perspective on immigration, all non-citizens and respondents who stated that they were part of an ethnic minority group were excluded from the analyses, leaving 32,748 respondents. We employed two weights that were delivered with the data; a population weight to take the size of each country into account and a design weight to correct for non-response bias.

### Variables

On the country level, ethnic diversity within a country was added to the data. Ethnic diversity was operationalized with *international migrant stock* data derived from The World Bank, which is the number of inhabitants born in a country other than that in which they live (including refugees). We calculated percentages of international migrant stock to take into account what proportion this number bears to the population of the country. Since the ESS-1 dates from 2002, with some fieldwork already in 2001, the international migrant stock dates from the year 2000, which was just before the data for the ESS-1 was collected.

On the individual level, two individual-level independent variables were also included: *Cross-group friendship* was measured with the following item: “Do you have any friends who have come to live in [country] from another country?” (1 = *no none at all*, 2 = *yes a few*, 3 = *yes several*). Since we expected to find the largest decrease in anti-immigrant sentiments for respondents who had cross-group friendships as compared to no cross-group friendships it was converted into a dummy variable (0 = *no immigrant friends*, 1 = *immigrant friends*). *Generalized trust* was measured with the following three items on an eleven-point Likert scale; i.e. “Generally speaking, would you say that most people can be trusted, or that you can’t be too careful in dealing with people?” (0 = *you can’t be too careful*, 10 = *most people can be trusted*), “Do you think that most people would try to take advantage of you if they got the chance, or would they try to be fair?” (0 = *most people would try to take advantage of me*, 10 = *most people would try to be fair*), and “Would you say that most of the time people try to be helpful or that they are mostly looking out for themselves?” (0 = *people mostly look out for themselves*, 10 = *people mostly try to be helpful*). Analysis shows that the internal coherence of the scale is more than acceptable, α_all countries_ = .77. Also, previous studies offer evidence for the measurement equivalence of generalized trust across European societies [47–49].

Moreover, a wide range of demographic variables were included in ESS-1. The following variables were included to control for several potentially spurious effects. First, *gender* was included as a control variable, because women tend to have more tolerant attitudes toward immigrants [50]. Second, *age* was calculated based on the respondent’s year of birth and was included because older respondents are generally more negatively biased toward immigrants [50]. Third, *years of education* was measured with “How many years of full-time education have you completed?” for comparability between countries. Fourth, we included income satisfaction as a proxy for household income, because of the large number of missing answers on the question about actual household income. *Income satisfaction* was measured with “Which of these descriptions comes closest to how you feel about your household’s income nowadays?” on a 4-point scale (1 = *finding it very difficult on present income*, 4 *= living comfortably on present income*). Education and income satisfaction were included as control variables because people in more vulnerable positions in the social system (i.e. low income, low education level) are more likely to hold negative attitudes toward immigrants [5,51,52]. Last, we controlled for religiosity, because in some cases it has been linked to negative intergroup attitudes [17,53]. *Religiosity* was measured with “Apart from special occasions such as weddings and funerals, about how often do you attend religious services nowadays?” on a 7-point scale (1 = *never*, 2 = *less often*, 3 = *only on special holy days*, 4 = at least once a month, 5 = once a week, 6 = *more than once a week*, and 7 = *every day*).

The dependent variable *anti-immigrant sentiments* was measured with three items on eleven-point Likert scales, i.e. “Would you say it is generally bad or good for a [country]’s economy that people come to live here from other countries?” (0 = *bad for the economy*, 10 = *good for the economy)*, “Would you say that [country]’s cultural life is generally undermined or enriched by people coming to live here from other countries?” (0 = *cultural life undermined*, 10 = *cultural life enriched)*, and “Is [country] made a worse or a better place to live by people coming to live here from other countries?” (0 = *worse place to live*, 10 = *better place to live)*. Afterwards, the three statements were reverse coded in which higher scores reflected higher levels of anti-immigrant sentiments to aid interpretation of the results. This three-item scale is considered as a solid and one-dimensional measurement of anti-immigrant sentiments [5]. Principal component analyses were conducted for each country separately to examine whether these three statements formed an equally coherent scale for each country, α_all countries_ = .80.

An overview of the range, means, and standard deviations of these variables is provided in Table 1.

**Table 1:** Descriptive statistics of variables used in the analysis.

	Min.	Max.	Mean/proportion	SD
*Country-level variable*				
International migrant stock	2.14	32.27	8.93	5.88
*Individual-level variables*				
Anti-immigrant sentiments	0	10	4.87	1.95
Generalized trust	0	10	5.16	2.00
Cross-group friendship	0	1	0.45	
Gender	0	1	0.53	
Age	14	110	47.09	18.28
Years of education	0	40	11.80	4.00
Religiosity	1	7	2.66	1.56
Income satisfaction	1	4	3.09	0.81

*Source*: ESS-1; The World Bank.

### Plan of analysis

The data was analyzed as follows. As a first step, mean levels of the main variables were examined per country, i.e. anti-immigrant sentiments, international migrant stock, cross-group friendships, and generalized trust. Next, these variables were analyzed by means of multilevel analysis to take into account the nested structure of the data and to control for possible confounding variables. Preceding the multilevel analysis, all variables (except for the dichotomous variables and the dependent variable) were grand mean centered. The multilevel models were organized into four stages. In Model 1, we ran an analysis using the demographic control variables (gender, age, years of education, religiosity, and income satisfaction) and international migrant stock to examine the relationship of ethnic diversity with anti-immigrant sentiments (H4). In Model 2, we added in cross-group friendship. In Model 3, we then added in generalized trust to examine its relation with anti-immigrant sentiments (H1). In addition, comparing Model 2 and 3 allows us to test how much of that relationship is accounted for by cross-group friendship, i.e. how much of the variance in anti-immigrant sentiments is uniquely explained by generalized trust (H2). In Model 4, we tested an individual-level interaction between generalized trust and cross-group friendship to examine whether the relation between generalized trust and anti-immigrant sentiments is dependent upon cross-group friendship (H3). The results for the steps discussed in this plan of analysis are presented in the results section below.

## Results

**Fig 1:**
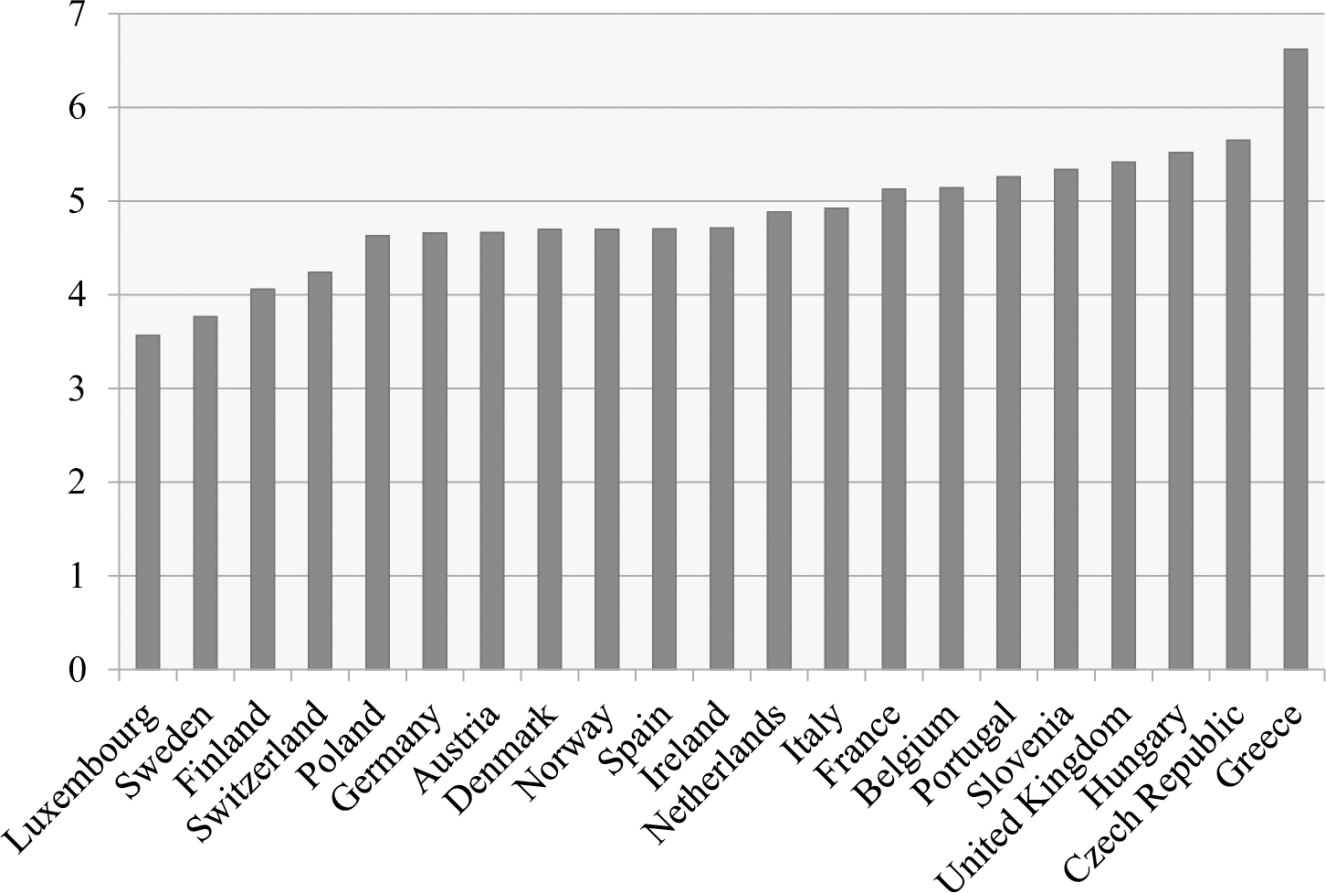
Country means for anti-immigrant sentiments (10 = *very high level of anti-immigrant sentiments*).

An overview of the country means for anti-immigrant sentiments is presented in Fig 1. As can be observed, the level of anti-immigrant sentiments was lowest in the Scandinavian countries, and highest in Greece.

Table 2 illustrates the percentage of international migrant stock and the mean levels of cross-group friendships and generalized trust in a country. It appeared that the countries in which there were negative attitudes toward immigrants are not necessarily the countries that have the highest levels of ethnic diversity. It is noticeable, however, that citizens in countries with high levels of anti-immigrant sentiments also report less generalized trust and cross-group friendships. Individual-level correlations revealed a significant negative relationship between having cross-group friendships and anti-immigrant sentiments, *r* = -.27, *p* = < .001. Moreover, there was a significant negative relationship between generalized trust and anti-immigrant sentiments, *r* = -.32, *p* = < .001. However, we noticed a very low correlation between generalized trust and having immigrant friends, *r* = .10, *p* = < .001. This could suggest that even though both cross-group friendship and generalized trust are negatively related to anti-immigrant sentiments, they are not necessarily related to each other.

**Table 2:** Percentage of international migrant stock, percentage of respondents having immigrant friends, and mean levels of generalized trust in 21 European societies.

Country	Percentage of international migrant stock	Percentage of people having immigrant friends	Mean level of generalized trust
Poland	2.14	43	3.79
Finland	2.85	41	6.34
Hungary	2.91	30	4.30
Italy	3.73	35	4.40
Spain	4.35	35	4.88
Czech Republic	4.42	30	4.40
Portugal	6.17	43	4.32
Norway	6.65	54	6.54
Greece	6.70	29	3.41
Denmark	6.95	46	6.85
United Kingdom	8.13	43	5.37
Belgium	8.57	44	4.98
Slovenia	8.77	52	4.30
Netherlands	9.96	41	5.74
Ireland	10.11	42	5.80
France	10.31	65	4.90
Sweden	11.19	67	6.26
Germany	12.14	46	5.09
Austria	12.44	50	5.27
Switzerland	21.75	73	5.79
Luxembourg	32.27	74	5.19

*Note*: The international migrant stock was derived from The World Bank, 2000.

Following preliminary analyses in SPSS 22, the xtmixed procedure in Stata 13 with full maximum likelihood estimation and robust standard errors was used to analyze the data. First, a null model (intercept-only model) with the dependent variable anti-immigrant sentiments was estimated. Based on the null model, the intraclass correlation (ICC) was calculated, which indicated that the percentage of variance at the country level indeed called for multilevel techniques of analysis; 11.34%. Substantively, this means that countries indeed differed with regard to anti-immigrant sentiments. Next, multilevel analyses were performed in four stages as was outlined in the plan of analysis. The results are shown in Table 3.

**Table 3:** Multilevel regression models of ethnic diversity, cross-group friendship, and generalized trust predicting anti-immigrant sentiments.

	Model 1		Model 2		Model 3		Model 4	
	B	Robust S.E.	B	Robust S.E.	B	Robust S.E.	B	Robust S.E.
International migrant stock	-.040***	.011	-.030**	.012	-.026*	.013	-.024	.015
Cross-group friendship			-.640***	.038	-.612***	.035	-.602***	.033
Generalized trust					-.227***	.012	-.230***	.013
Cross-group friendship * generalized trust							.007	.015
*Control variables*								
Gender	.029	.040	.011	.037	.030	.032	.032	.032
Age	.001	.001	-.002	.001	-.001	.001	-.001	.001
Years of education	-.116***	.010	-.103***	.010	-.093***	.009	-.094***	.009
Religion	-.000	.015	-.003	.014	.012	.013	.012	.013
Income satisfaction	-.259***	.023	-.255***	.021	-.177***	.022	-.177***	.023
Intercept	4.878***	.119	5.175***	.117	5.154***	.109	5.155***	.109
*Variance components*								
Variance country level	0.275		0.257		.213		.222	
Variance individual level	3.033		2.945		2.795		2.791	
Variance random slope							.019	
*Model fit*								
R^2^ country level	.363		.405		.507		.486	
R^2^ individual level	.102		.128		.172		.174	

*Note*. Entries are the result of a multilevel regression analysis of the ESS 2002 dataset.

Sign.

*** *p* < .001

** *p* < .01

* *p* < .05.

Model 1 revealed that international migrant stock significantly predicted a decrease in anti-immigrant sentiments, *b* = -.04, *p* < .001. In more ethnically diverse countries there was less prejudice against immigrants. The same model was tested with inflow of foreigners derived from the Organisation for Economic Co-operation and Development (OECD) instead of international migrant stock, with similar results, *Z*_*inflow*_ = -2.82, *p* = .005 and *Z*_*stock*_ = -3.54, *p* < .001. This result in part supports H4 that ethnic diversity does not predict an increase in anti-immigrant sentiments. However, the evidence also goes one step further by showing that in fact ethnic diversity is *negatively* related to anti-immigrant sentiments. Hence, ethnic diversity does not lead people to ‘hunker down’ as anti-immigrant sentiments are clearly lower in more diverse societies. Self-evidently, this does not yet imply any causality as it is also likely that immigrants are more strongly attracted to societies with lower levels of anti-immigrant sentiments.

Model 2 demonstrated that cross-group friendship significantly predicted a decrease in anti-immigrant sentiments, *b* = -.64, *p* < .001. Also, Model 3 revealed that generalized trust significantly predicted a decrease in anti-immigrant sentiments, *b* = -.23, *p* = < .001. This is in line with H1 and shows that immigrants too are included in judgments of generalized trust. Moreover, it can be observed that including generalized trust in the analysis considerably strengthened the explained variance of the model. Indeed, all other variables kept their level of significance, indicating that generalized trust contributed to the theoretical strength of the analysis. The b-coefficient of cross-group friendship, too, remained almost identical, implying that generalized trust did not play a role in the attitudinal effect of cross-group friendships. Subsequently, Model 4 demonstrated that generalized trust was also not a moderator for the relation between cross-group friendship and anti-immigrant sentiments. Thus, support is found for H2 and H3: Cross-group friendships and generalized trust are not associated (H2), and the negative relation between generalized trust and anti-immigrant sentiments is not dependent upon cross-group friendships (H3).

## Discussion and conclusion

The current study has several important findings. First, in line with expectations, results showed that both generalized trust and cross-group friendships were substantially negatively related to the occurrence of anti-immigrant sentiments. These findings are compatible with earlier research efforts showing that generalized trust has indeed a wide circle of trusted others in which immigrants are also included [16]. Trusting actors extend this positive worldview generally to members of society, including the ones that do not share the same ethnic or cultural characteristics. Second, we can clearly observe that generalized trust and cross-group friendship operated independently from another. So, it was not the case that more trusting respondents were more likely to form more cross-group friendships or that, vice versa, cross-group friendship leads to the development of generalized trust. While in some of the previous literature a positive relationship was found between intergroup contact and other forms of trust, such as outgroup trust [19–21], in the current research we did not find a similar relationship for generalized trust. Future research could help us explain the difference between outgroup trust and generalized trust in this regard. Third, the results confirm earlier research by showing that ethnic diversity does not lead to higher levels of anti-immigrant sentiments. In fact the opposite phenomenon is more likely to occur as there is much more diversity in countries with low levels of anti-immigrant sentiments.

A caveat to be noted here is that we only control for cross-sectional relations, so it is quite well possible that either immigrants are more strongly attracted to countries with lower levels of anti-immigrant sentiments, or that other, for example, economic or cultural elements are related both to the attractiveness toward immigrants as to levels of anti-immigrant sentiments [54]. Moreover, the generalizability of the findings may be limited by the use of less recent data from 2002. Studies have suggested that anti-immigrant sentiments may fluctuate over time [5,55–57], which means that current levels of anti-immigrant sentiments may be different than was reported in this research. Similar changes or increases can be expected for levels of ethnic diversity. However, there is no indication that the *nature* of the examined relations between ethnic diversity, generalized trust, and cross-group friendships on the one hand, and anti-immigrant sentiments on the other hand, have changed over time [5]. Last, it is important to bear in mind that the current study used the proportion or inflow of inhabitants born in a country other than that in which they live (including refugees) as an operationalization of ethnic diversity, which is in line with much research. However, there are many other possible operationalizations of ethnic diversity. For instance, in future investigations, ethnic diversity could be considered more broadly and also examine religious diversity or linguistic diversity. Particularly linguistic diversity may impede the prejudice-reducing effects of intergroup contact but may leave the negative relation between generalized trust and anti-immigrant sentiments intact. In addition, it might be worthwhile for future research to examine the role of *change* in ethnic diversity for anti-immigrant sentiments. A sudden increase in ethnic diversity may evoke perceptions of intergroup threat that drowns out the opportunity for intergroup contact [22].

The main finding of the current analysis therefore is that the negative relationship between generalized trust and anti-immigrant sentiments is not dependent upon cross-group friendships. In trying to understand the effects of generalized trust, there is at least one mechanism that we can already exclude. It is not the case that more trusting people have an easier time to develop cross-group friendships, and that as a result of these friendships, they develop more positive attitudes toward immigrants. If this was the case, both effects would not operate independently from another. Apparently, generalized trust has a much more direct relation with anti-immigrant sentiments. Individuals with high levels of trust also include immigrants in their broad, encompassing conception of the ‘others’ in society. Given the broad radius of the generalized trust concept, it indeed makes no sense to exclude a substantial part of the population of one’s society from a general expression of trust. This does imply that the relation between generalized trust and anti-immigrant sentiments to some degree might even be tautological, as they can be interpreted as two opposite formulations of the same concept. While generalized trust expresses the idea that all members of society should be included, anti-immigrant sentiments explicitly starts from the assumption that at least a substantial part of the population should not belong to society as a whole. The strength of generalized trust apparently is a form of generalization, beyond the confines of actual experiences with immigrants, and in this case the generalization ran in exactly the opposite direction as the generalization that is also present in anti-immigrant sentiments. Both trusting and non-trusting people are equally likely to be confronted with some of the more negative consequences of increased diversity, such as increased competition in the labor market. A difference, however, might be that while those less trusting generalize these kind of experiences into a general negative attitude, those with higher generalized trust levels–even with exactly the same experiences–do not embark on this kind of generalization.
